# Clinical efficacy and safety of Qingshu Yiqi decoction as a complementary and alternative therapy for COVID-19 with Delta variant: A protocol for systematic review and meta-analysis

**DOI:** 10.1097/MD.0000000000028184

**Published:** 2021-12-17

**Authors:** Yu Xia, Weiyu Qi, Xin Li, Yuying Yang, Jianzhong Cao

**Affiliations:** a Hunan University of Chinese Medicine, China; b Hunan Provincial Key Laboratory of Diagnostics in Chinese Medicine, Hunan University of Chinese Medicine, Xueshi Road, Yuelu District, Changsha, Hunan, China.

**Keywords:** complementary and alternative medicine, COVID-19, delta variant, meta-analysis, Qingshu Yiqi decoction

## Abstract

**Background:**

Qingshu Yiqi decoction combined with general western medicine are important and prevalent recently treatment method of corona virus disease 2019 (COVID-19) with Delta variant, but the efficacy and safety were not clear. This meta-analysis aims to clarify their clinical efficacy and safety thus to provide evidence for clinical application.

**Methods:**

We searched Chinese databases CNKI, Wanfang, VIP and English databases PubMed, Cochrane Library, Embase for the current study. The retrieval time was from the establishment to November, 2021. Literature quality was evaluated according to the bias risk assessment criteria of Cochrane Collaboration network. RevMan 5.3 and Stata 12.0 were used to perform this research.

**Results:**

The efficacy and safety of Qingshu Yiqi decoction combined with western medicine for COVID-19 with Delta variant were evaluated in terms of total effective rate, TCM syndrome score, negative conversation rate of viral nucleic acid, disappearance rate of clinical symptoms (such as fever, cough, and fatigue), CT improvement, white blood cell (WBC), lymphocyte (LYM) and adverse reaction rate.

**Conclusion:**

This study provides reliable evidence-based support for the clinical efficacy and safety of Qingshu Yiqi decoction as a complementary and alternative therapy for COVID-19 with Delta variant.

**PROSPERO registration number:**

CRD42021271606.

## 1 Introduction

The outbreak of an unknown pneumonia was diagnosed in Wuhan, China, in December, 2019.^[1]^ The etiology responsible for the pneumonia was soon determined as a novel coronavirus, and the full viral genome was sequenced and showed 96% similar to a bat coronavirus SARS-CoV-RaTG13 in January, 2020.^[2,3]^ The virus was initially named as 2019-nCoV and changed formally to SARS-CoV-2 by the Coronavirus Study Group of the International Committee on Taxonomy of Viruses.^[4]^ SARS-CoV-2 could infect the lower respiratory tract and cause mild to severe pneumonia in humans. Corona virus disease 2019 (COVID-19) named by the World Health Organization (WHO),^[5]^ refers to the pneumonia caused by SARS-CoV-2 infection. The main manifestations of COVID-19 are fever, dry cough and fatigue. A small number of patients have upper respiratory and digestive tract symptoms such as stuffy nose, runny nose and diarrhea. Most severe cases developed dyspnea after 1 week, and even rapidly progressed to acute respiratory distress syndrome, septic shock, refractory metabolic acidosis, coagulation dysfunction, and multiple organ failure.

Delta is the mutant strain of SARS-CoV-2. It was first discovered in India in October 2020.^[6]^ In May, 2021, WHO named the mutant strain B.1.617.2 as the “Delta” variant. This variant was identified as one of the drivers of the second wave of outbreaks in India. On 25 June, 2021, WHO stated that transmission of Delta variant has been detected in 92 countries and territories worldwide.^[6,7]^ Delta variant, the most transmissible variant ever discovered, is spreading rapidly among unvaccinated people.^[8]^ Delta variant, which is more human-affiliative and heat-resistant, is characterized by greater transmissibility, high viral load, high pathogenicity, and possible immune escape, but the vaccine still provides protection.^[9,10]^ The clinical symptoms of Delta variant were not significantly different from those of previous strains, and they were all familiar symptoms such as fever, dry cough and fatigue.

For the treatment of COVID-19, the strategy of new use of old drugs is mainly adopted to screen candidates for anti-SARS-CoV-2 and suppression of excessive immune response. Specific proteins and host enzymes involved in each step in the life cycle of the virus provide potential drug targets for treatment. These potential drug targets include non-structural proteins (such as 3-chymotrypsin like protease, papain like protease, helicase, and RNA-dependent RNA polymerase), structural proteins (such as S protein), and accessory proteins.^[11,12]^ However, up to now, clinical treatment of COVID-19 in western medicine is mostly symptomatic treatment, and there is still no specific antiviral drugs.

From the perspective of traditional Chinese medicine, the current outbreak still falls under the category of epidemic disease. The diagnosis is “dampness-toxin plague” as in previous COVID-19 cases,^[13,14]^ and the core pathogenesis remains unchanged. The pathogenic characteristics of Delta variant are fast onset, large viral load and strong infectivity. According to the etiology of traditional Chinese medicine, it belongs to “blazing toxin”.^[15]^ From the season of onset, the outbreak of the epidemic last year was mainly in winter and spring. The climate was cold, and the characteristics of dampness-toxin-cold plague were more prominent. Cold is Yin evil, and the main manifestations of the patients were that the fever symptoms were not obvious and the progress of the disease was relatively slow. If the treatment was not timely, it generally took 7 to 14 days to develop into severe or critical case.^[16]^ The epidemic caused by Delta variant occurs in summer, which is easy to mix heat and dampness. Summer-heat is Yang evil, and the toxic-heat of dampness toxin caused by Delta variant is more intense, mixed with heat evil, and the heat will change more rapidly; Heat evil is easy to consume the healthy qi of the human body and damage the body fluid. Patients are prone to be qi deficiency. Therefore, the characteristics and clinical symptoms of the patients are different from last year. Combined with the characteristics of the patients, the time from infection to clinical discomfort was shorter than that of last year, the disease progressed faster, and the patients’ fever symptoms were prominent, which was in line with the evolution characteristics of the pathogenesis of “dampness and heat”.^[17,18]^

In terms of traditional Chinese medicine treatment, we still refer to the “three drugs and three prescriptions” in the eighth edition of the national diagnosis and treatment scheme.^[16]^ However, when using the recommended prescription, we should make appropriate adjustments according to the characteristics of patients, especially pay attention to increasing the use of heat clearing and aromatic detoxification drugs. Qingshu Yiqi decoction is a summer-heat clearing prescription, which has the effects of clearing summer-heat and tonifying qi, nourishing yin and generating fluid. It is mainly used for consumption of both fluid and qi caused by summer-heat.^[19]^ In clinical application, the key points of syndrome differentiation are fever and sweating, thirst and upset, fatigue and weak breath, and the pulse is feeble and rapid. Preliminary research shows that western medicine combined with traditional Chinese medicine has a certain effect on COVID-19 and has achieved certain results.^[20–22]^ However, due to the limitations of the study and the small sample size, it has certain limitations in guiding the clinical application of Qingshu Yiqi decoction combined with general western medicine in the treatment of COVID-19 with Delta variant. Therefore, this current meta-analysis aims to systematically evaluate and analyze its clinical efficacy and safety, so as to provide evidence-based medical reference for COVID-19 with Delta variant.

## 2 Methods

### 2.1 Study registration

The protocol of this systematic review and meta-analysis refers to the guidelines of the Preferred Reporting Items for Systematic Reviews and Meta-Analyses protocols (PRISMA-P).^[23]^ This protocol has been registered on the PROSPERO (registration number: CRD42021271606).

### 2.2 Ethics

Since the data of this review were derived from published literature, ethical approval is not required.

### 2.3 Inclusion criteria

#### 2.3.1 Types of studies

Only randomized controlled trials (RCTs) of Qingshu Yiqi decoction combined with general western medicine in the treatment of COVID-19 with Delta variant will be included, regardless of publication or region, but language will be restricted to Chinese and English.

#### 2.3.2 Participants

The research subjects were COVID-19 with Delta variant patients, without gender, age and race limitations.

#### 2.3.3 Interventions

The experimental groups: Qingshu Yiqi decoction combined with general western medicine; The control groups: General western medicine (In line with the COVID-19 Diagnosis and Treatment Program issued by the National Health Commission).

#### 2.3.4 Outcomes

The primary outcomes included: Total effective rate, TCM syndrome score and negative conversation rate of viral nucleic acid. The TCM syndrome score scale is composed of 23 items to evaluate. Each item is worth 1 to 4 points, so the total TCM syndrome scores that each patient earned ranged from 23 (best) to 72 (worst).

The secondary outcomes included: Disappearance rate of clinical symptoms (such as fever, cough, and fatigue), CT improvement, WBC, LYM and adverse reaction rate.

### 2.4 Exclusion criteria

1. Repeated publications.
2. The data is incomplete and cannot be extracted for analysis.
3. Unclear outcome.
4. Animal experiments, cell experiments and the review literature.
5. Case reports.
6. The control group was combined with other treatments.

### 2.5 Information sources and literature search

Based on the Preferred Reporting Items for Systematic Reviews and Meta-Analyses guidelines, the meta-analysis is performed. Databases searched included Chinese databases CNKI, Wanfang, VIP and English databases PubMed, Embase and Cochrane Library. The retrieval time is from the establishment to August 2021. Chinese search terms: “COVID-19 (xin xing guan zhuang bing du fei yan)” OR “SARS-CoV-2 (xin xing guan zhuang bing du)” AND “Delta variant (de er ta bian yi zhu)” AND “Qingshu Yiqi decoction (qing shu yi qi tang)” AND “combined therapy (lian he zhi liao)” AND “clinical trial (lin chuang shi yan)” OR “randomized controlled trial (sui ji dui zhao shi yan)”; English search terms: “coronavirus disease 2019” OR “COVID-19” OR “SARS-CoV-2” AND “Delta variant” AND “Qingshu Yiqi decoction” OR “Qingshu Yiqi Tang” OR “Qingshu Yiqi Soup” AND “combined therapy” OR “combined with” AND “clinical trial” OR “randomized controlled trial”. Taking PubMed as an example, the search strategy is listed in Table 1.

**Table 1:** Search strategy for PubMed database.

Number	Search items
#1	Coronavirus disease 2019
#2	COVID-19
#3	SARS-CoV-2
#4	#1 OR #2 OR #3
#5	Delta variant
#6	Qingshu Yiqi decoction
#7	Qingshu Yiqi Tang
#8	Qingshu Yiqi Soup
#9	#6 OR #7 OR #8
#10	combined therapy
#11	combined with
#12	#10 OR #11
#13	clinical trial
#14	randomized controlled trial
#15	#13 OR #14
#16	#4 AND #9 AND #12 AND #15

### 2.6 Literature selection

**Figure 1:**
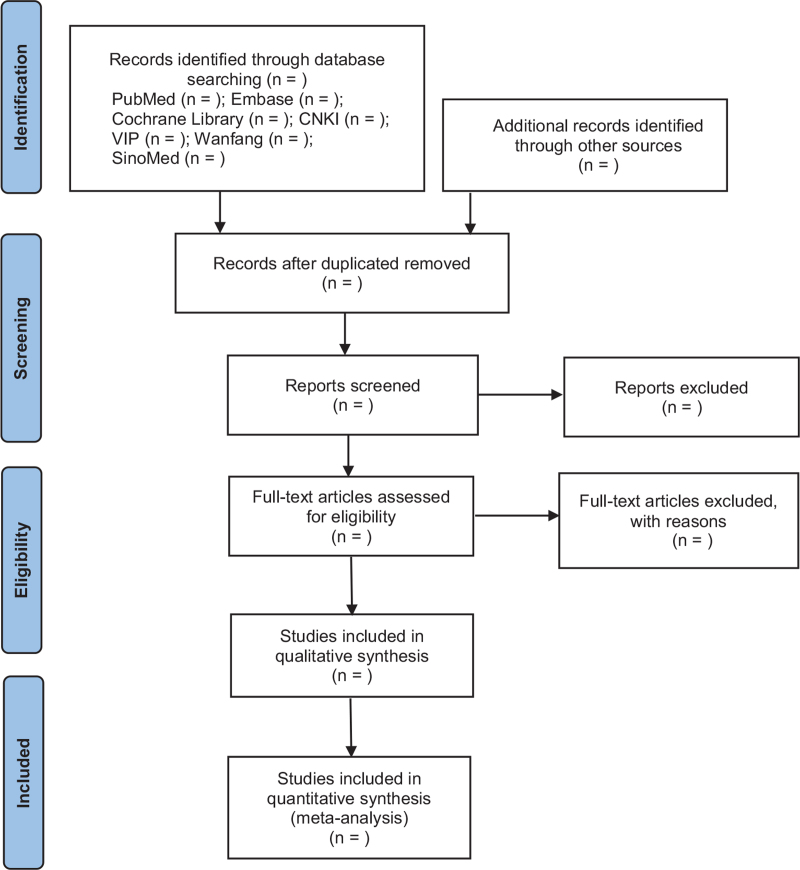
Flow chart showing literature screening process.

Data extraction and quality assessment are conducted by two researchers independently according to the screening criteria, then cross-checked the data. If there are conflicts of opinions, resolve them through collective discussion. In the process of literature screening, the literature with irrelevant titles are excluded, and the abstracts and full texts should be further read to determine the final included literature. The process of literature screening is shown in Figure 1.

### 2.7 Data extraction and quality assessment

1. The basic information contained: first author, year of publication, sample size, interventions, and course of treatment.
2. Bias risk assessment: including random grouping methods, allocation concealment, blinding methods, incomplete data, and each is rated as either “high risk”, “low risk” or “unclear”.
3. Literature quality is evaluated according to the bias risk assessment criteria of Cochrane Collaboration network.

### 2.8 Statistical analysis

RevMan 5.3 and Stata 12.0 are used for this meta-analysis. The calculation of the dichotomous variable is expressed by odds ratio (OR), and mean difference (MD) or standardized mean difference (SMD) is calculated for the continuous variable. 95% confidence interval (95% CI) is expressed for the numerical value. Heterogeneity is evaluated by I^2^ and chi-square tests. The fixed-effects model is used for analysis if I^2^ ≤ 50%, the random-effects model is used if I^2^ > 50%. The results are shown in the forest plot. For all outcomes, publication bias is assessed by Egger and Begg tests.

### 2.9 Sensitivity evaluation

The sensitivity of the meta-analysis is evaluated by changing the effect model, and the changed OR and MD (SMD) value are used for the sensitivity analysis.

### 2.10 Grading the quality of evidence

The Grading of Recommendation, Assessment, Development and Evaluation (GRADE) system^[24]^ will be used to appraise the quality of evidence from the researches obtained. The levels of it will be divided into high, moderate, low, very low.

## 3 Discussion

Delta variant and SARS-CoV-2 all belong to coronavirus. Delta variant belongs to the mutant strain, with stronger toxicity, shorter incubation period and strong infectivity. Delta variant has the greatest risk of asymptomatic infection and is easy to develop into severe disease, which should be paid attention to. However, both the Delta variant and SARS-CoV-2 belong to the epidemic disease in traditional Chinese medicine. Its characteristics are acute onset, rapid transmission, and its prevention is more important than treatment.

From the perspective of traditional Chinese medicine, epidemic toxin is a kind of epidemic pathogenic factor that is out of time. After invading the human body, it changes in many ways. It turns heat, torments the yin fluid of the lung, consumes the lung fluid, and results in disturbance of diffusion and downbearing. Lung and spleen belong to the taiyin meridian. Among the five phases, lung belongs to gold, spleen belongs to earth, and disorder of the child organ affects the mother organ, thus leading to spleen deficiency and dampness, which eventually leads to qi and yin deficiency in the recovery period. Qingshu Yiqi decoction is derived from the *Treatise on the Spleen and Stomach.*^[25]^ It can treat the deficiency of qi and yin, with heat dampness. It has the effects of clearing qi aspect heat, clearing heat and generating fluid, tonifying qi and harmonizing stomach. In this prescription, Dangshen (*Codonopsis pilosula*) and Huangqi (*Astragalus membranaceus*) replenish qi and secure the exterior. Cangzhu (*Rhizoma atractylodis*) and Baizhu (*Atractylodes macrocephala*) strengthen the spleen and dry dampness. Huangbo (*Phellodendron chinense*), Maidong (*Ophiopogon japonicus*) and Wuweizi (*Schisandra chinensis*) purge fire and promote the secretion of saliva. Chenpi (*Pericarpium citri reticulatae*), Qingpi (*pericarpium citri reticulatae viride*) and Zexie (*Rhizoma alismatis*) regulate qi and excrete dampness. Danggui (*Angelica sinensis*) nourishes blood and yin. Shengma (*Rhizoma cimicifugae*) and Gegen (*Pueraria Lobata*) resolve the flesh and upbear the clear. Gancao (*Glycyrrhiza*) moderates the herbs. The combination of various herbs plays the function of clearing summer-heat, dissipating dampness, tonifying qi and generating fluid. Modern pharmacological research has also confirmed that Huangqi (*Astragalus membranaceus*) can improve the ability of anti-stress response and anti-inflammatory. Gancao (*Glycyrrhiza*) can maintain hormone balance in the body. Danggui (*Angelica sinensis*) and Gegen (*Pueraria lobata*) also have anti-inflammatory effects.^[26]^

However, there are some limitations of this study. The limited sample size may influence the comprehensiveness of the whole results. The complexity of dosages and components increased the difficulty of final interpretation of the results. In addition, slight publication bias and the low quality of RCTs of the included studies may diminish the power of this meta-analysis. Therefore, more well-designed, rigorously conducted and RCTs of high quality are needed to verify the efficacy and safety of Qingshu Yiqi decoction as a complementary and alternative treatment of COVID-19 with Delta variant.

## Author contributions

**Conceptualization:** Yu Xia.

**Data curation:** Yu Xia, Xin Li.

**Formal analysis:** Yuying Yang.

**Funding acquisition:** Jian-zhong Cao.

**Investigation:** Weiyu Qi.

**Software:** Yu Xia, Weiyu Qi.

**Supervision:** Xin Li.

**Writing – original draft:** Yu Xia.

**Writing – review & editing:** Jian-zhong Cao.
